# Guy’s Hospital

**Published:** 1852-11

**Authors:** 


Guy's Hospital. *Consulting Physician*—Dr. Bright. *Physicians*—Drs. Addison, Babington, and Barlow. *Assistant Physicians*—Drs. Hughes, Owen Rees, Golding Bird, and Gull. *Surgeons*—Messrs. B. Cooper, Cock, and Hilton. *Assistant Surgeons*—Messrs. Birkett and Poland. *Obstetric Physicians*—Drs. Lever and Oldham. *Surgeon Dentist*—Mr. Bell. *Surgeon of the Eye Infirmary*—Mr. France. *Apothecary*—Mr. Stocker.

Medical and Surgical School. *Winter Session.*—Anatomy, Descriptive, Surgical, and Pathological, Messrs. Hilton and Birkett. Physiology, Dr. Gull. Demonstrations on Anatomy, Mr. Callaway and Dr. Habershon. Chemistry, Dr. Taylor. Medicine, Dr. Addison. Surgery, Mr. Bransby Cooper. Natural Philosophy, Dr. Lloyd. Moral Philosophy, Rev. T. H. Bullock. Clinical Medicine, Drs. Addison, Babington, and Barlow. Clinical Surgery, Messrs. Bransby Cooper, Cock, and Hilton. Clinical Lectures on Midwifery and Diseases of Women, Drs. Lever and Oldham. *Summer Session.*—Demonstrations on Cutaneous Diseases, Dr. Addison. Materia Medica, Drs. Golding Bird and Rees. Clinical Lectures, Drs. Hughes, Owen Rees, and Golding Bird. Regional Anatomy, Mr. Hilton, or Mr. Birkett. Dental Surgery, Messrs. Bell and Salter. Pathological Anatomy, Dr. Lloyd. Midwifery, Drs. Lever and Oldham. Medical Jurisprudence, Dr. Taylor. Comparative Anatomy, Drs. Gull and Habershon. Ophthalmic Surgery, Mr. France. Botany, Mr. Johnson. Practical Chemistry, Dr. Odling.

